# An evaluation of cases of disorders of sex development related to *SRD5A2*

**DOI:** 10.1007/s12020-025-04252-5

**Published:** 2025-05-17

**Authors:** Can Celiloglu, Ihsan Turan, Leman Damla Kotan, Ayşe Merve Cimen, Semine Ozdemir Dilek, Bilgin Yuksel

**Affiliations:** 1 Cukurova University Faculty of Medicine, Department of Pediatric Endocrinology, Adana, Turkey; 2 Adana City Training and Research Hospital, Department of Pediatric Endocrinology, Adana, Turkey

**Keywords:** 46, XY disorders of sex development, SRD5A2 gene, Gonadectomy, Gender assignment

## Abstract

**Purpose:**

Pathogenic variants in *SRD5A2* are a common cause of 46,XY disorders of sex development (DSDs). The aim of this study is to present the clinical, laboratory, and genetic characteristics of patients diagnosed with SRD5A2-associated 46,XY disorders of sex development (DSD), along with any decisions made regarding sex assignment. Furthermore, it also highlights the challenges encountered in sex assignment and the potential influence of social factors on how families adapt to these decisions.

**Methods:**

This retrospective, single-center study analyzed 29 DSD cases with 46,XY karyotypes, all of which were found to carry *SRD5A2* variants.

**Results:**

The majority of the patients initially presented with female sex assignment (86.2%). The study identified a predominance of homozygous *SRD5A2* variants (93.1%) with the most common variant being p.Ala65Pro. Gender assignment decisions were made for 25 cases, with male gender assignment in 80% of cases. The parents of three adolescent athlete patients did not comply with the male gender decision and one of these patients was supported as a girl after the age of 18, with a corrective operation being subsequently performed. No patients underwent a gonadectomy before the age of 18.

**Conclusion:**

This study emphasizes the challenges in sex assignment for *SRD5A2*-associated DSD. Some XY DSD patients may have difficulty adhering to medical team guidance because of the negative impact of the patients in their families. When necessary, gender assessment committees should reevaluate DSD cases from a current perspective and reconsider their decisions.

## Introduction

Male sexual development requires testosterone (T) and dihydrotestosterone (DHT) for the virilization of internal and external genitalia. The enzyme 5-alpha reductase (5α-R2), which is responsible for converting testosterone to the more potent dihydrotestosterone, is encoded by the *SRD5A2* gene, which maps to chromosome 2p23 [1]. 46,XY disorders of sex development (DSDs) are caused by abnormalities in more than 60 genes, with *SRD5A2* pathogenic variants being one of the most common causes [2, 3]. Biallelic pathogenic variants of the gene have been associated with the disease, and it has been suggested that the phenotype may be influenced by *SRD5A2* polymorphisms [4]. *SRD5A2*-associated disorders may present across a spectrum including complete female appearance, ambiguous external genitalia, micropenis, and hypospadias [5]. The genotype‒phenotype relationship cannot be established in *SRD5A2*-associated pathologies [6]. Traditionally, an elevated T/DHT ratio following basal or hCG stimulation may indicate pathologies resulting from *SRD5A2* pathogenic variants. However, this ratio may be low in genetically confirmed cases [3]. The T/DHT ratio may lead to diagnostic confusion, and its importance in the diagnostic process has been debated [3]. Genetic testing is a reliable method of confirming the diagnosis of disorders linked to SRD5A2.

Long-term follow-up cohorts mature the decision-making process regarding the disease. It has been reported that individuals with *SRD5A2* pathological variants, who were raised mostly as females approximately 20 years ago, felt male in approximately 60% of the cases during follow-up after an established assignment as female [7]. Currently, the support of *SRD5A2*-associated XY DSD cases as male is widely accepted, contrary to previous practices [8]. As a general approach, timely surgical interventions are known to contribute to patient prognosis. It is recommended that patients with cryptorchidism should undergo surgery before 18 months of age, and patients with hypospadias should undergo surgery at 1–2 years of age [9, 10]. Moreover, for *SRD5A2*-associated DSD patients, even if the external genitalia are completely female, follow-up and treatment approaches in a gonad-protective manner stand out. Early diagnosis in childhood is important for the timely provision of appropriate medical support for individuals with disorders of sex development.

While *SRD5A2*-associated conditions are among the most common XY disorders of sex development, the number of reports on the follow-up and sex assignments of these cases is insufficient. The pathogenic variants causing *SRD5A2*-associated conditions are not homogeneously distributed worldwide, with various variants being prominent in various ethnic groups. In this study, we present the clinical, laboratory, and genetic characteristics of patients diagnosed with SRD5A2-associated 46,XY disorders of sex development (DSD), along with the decisions made by the Multidisciplinary Gender Assessment Committee (GAC). Additionally, this study aims to present the challenges during the sex determination process of children from our region who are professional athletes and come from families in which the disease segregates.

## Methods

### Cases

This retrospective, single-center study analyzed 29 DSD cases with 46,XY karyotypes (all of which were found to carry *SRD5A2* variants), at our tertiary care center. Age at diagnosis, presenting complaints, consanguinity between parents, family history of disorders of sexual development (DSDs), gender at presentation and assigned gender, external genitalia score, Quigley score, hormonal profile, basal and/or stimulated T and DHT levels, and variant and segregation analysis results were assessed [11, 12]. All subjects underwent an hCG challenge test consisting of three intramuscular hCG injections on consecutive days at a daily dose that varied according to age (<1 year, 500 units; 1–10 years, 1000 units; >10 years, 1500 units). Blood samples were taken before the first dose and 24 h after the last dose.

### Molecular genetics

Genomic DNA was extracted from peripheral blood samples via various kits at different centers following standard procedures. The genetic analyses of 13 cases were performed via “next-generation sequencing”, whereas 16 cases were analyzed via Sanger sequencing. The genetic analysis method we chose was shaped by the laboratory facilities we had access to at the time.

All detected SRD5A2 variants were searched in the ClinVar (https://www.ncbi.nlm.nih.gov/clinvar/) and PubMed databases. The CADD score (https://cadd.gs.washington.edu/snv) was used to predict the effect of the variants in silico. The Genome Aggregation Consortium (gnomAD, http://gnomad.broadinstitute.org/) and the Turkish Variome dataset were used to determine the allelic frequencies for each variant [13]. VarSome (https://varsome.com/) was used to determine variant classification on the basis of the 2015 American College of Medical Genetics and Genomics and Association for Molecular Pathology guidelines (ACMG/AMP) [14].

## Results

### Demographic and clinical data

A total of 29 cases of DSD with a 46,XY karyotype and *SRD5A2* pathogenic variants from 17 families were analyzed. At the time of application, the initial sex assignment of the patients was 25 female (86.2%), and 4 male (13.7%). There were no cases whose diagnostic processes were initiated during the antenatal or neonatal period. The median age at presentation was 71 months (min: 6 max: 201 months). In the medical history of the patients, a horseshoe kidney was detected in one patient, and there were no malignant pathologies in any of the patients. The rate of consanguineous marriage was 89.6% (26/29).

The primary complaints at the time of application were gender ambiguity (*n* = 15), positive family history (*n* = 4), micropenis (*n* = 2), masculine behaviors (*n* = 2), inguinal swelling (*n* = 2), amenorrhea (*n* = 2), vaginal adhesions (*n* = 1) and short stature (*n* = 1) (see Table 1). Consanguinity between parents was declared in 26 cases (89.6%). In prepubertal cases (*n* = 21), the mean phallus length was found to be 1.4 cm, with a median Quigley score of 5. In patients diagnosed during puberty (*n* = 8), the median phallus length was found to be 3.75 cm, with a median Quigley score of 3.5.

**Table 1:** A Demographic and basic clinical characteristics of the *SRD5A2* patients (Prepeubertal cases); B Demographic and basic clinical characteristics of the *SRD5A2* patients (Pubertal cases)

A												
Family	Formal Gender	Age (Months)	Application complaint	Year of Admission	Quigley Score	Phallus length(cm)	T (ng/ml)	DHT (ng/ml)	T/DHT	Post-hcg T	Post-hcg DHT	Post-hcg T/DHT
A	f	134	PFH	2018	3	3.0	4.87	0.20	24.3	NA	NA	NA
	f	69	GA	2018	5	1.0	0.75	0.02	37.5	NA	NA	NA
	f	12	GA, cliteromegaly	2019	6	0.8	<0.1	0	NA	NA	NA	NA
	f	9	GA, PFH	2019	4	1.0	0.34	0.03	11.3	NA	NA	NA
	f	71	GA, groin swelling	2018	3	2.0	2.22	0.26	8.5	NA	NA	NA
C	m	39	Micropenis	2021	5	2.5	NA	NA	NA	NA	NA	NA
D	f	23	PFH	2014	6	0.5	0.53	0.08	6.6	2,12	0,113	18,7
	f	71	PFH	2014	5	1.0	<0.1	0.08	NA	0,26	0,072	3,6
	f	126	Short stature, male karyotype	2014	4	1.0	0.08	0.14	0.57	0,28	0,132	2,1
F	f	65	Vaginal adhesion	2020	6	0.5	<0.1	0.954	NA	NA	NA	NA
G	f	6	GA	2018	4	1.5	0.93	0.54	1.72	NA	NA	NA
H	f	6	GA	2021	5	0.5	1,78	0.08	22.2	NA	NA	NA
J	m	32	GA	2013	6	0.7	1.63	0.13	12.5	4.70	0.36	13.8
K	f	46	GA	2017	3	2.0	0.1	0	NA	0.45	0.005	90
L	f	82	Masculine behaviors	2021	4	1.5	0,03	NA	NA	NA	NA	NA
	f	76	Masculine behaviors	2021	5	2.0	0.13	NA	NA	NA	NA	NA
	f	62	GA	2021	5	1.5	0.13	NA	NA	NA	NA	NA
M	m	136	GA	2013	4	0.7	0.01	NA	NA	NA	NA	NA
N	f	11	GA	2019	5	3.0	<0,1	NA	NA	NA	NA	NA
O	m	32	Micropenis	2018	4	2.5	0	0.2	NA	NA	NA	NA
P	f	32	GA	2024	3	0.5	NA	NA	NA	NA	NA	NA

B													
Family	Formal Gender	Age (Months)	Application complaint	Year of Admission	Puberty phase	Quigley Score	Phallus length(cm)	T (ng/ml)	DHT (ng/ml)	T/DHT	Post-hcg T	Post-hcg DHT	Post-hcg T/DHT
A	f	201	GA	2005	5	4	4.0	4	0.21	19	NA	NA	NA
B	f	165	Swelling in the groin	2022	3	3	4.0	3.47	0.13	26.6	NA	NA	NA
D	f	191	PFH	2014	2	4	3.5	3.42	0.35	9.71	11,6	0,8	14,5
E	f	156	GA	2017	5	3	4.5	5.24	0.89	5.88	NA	NA	NA
G	f	169	GA	2018	4	5	3.0	1.58	0.03	52.9	NA	NA	NA
I	f	201	Amenorrhea	2022	3	3	2.0	2.08	0.39	5.3	NA	NA	NA
P	f	126	Groin swelling	2024	2	4	1.5	0.74	0,07	10.5	NA	NA	NA
R	f	200	Amenorrhea	2024	5	2	4.0	5.56	0.15	37	NA	NA	NA

*f* female, *m* male, *PFH* positive family history, *GA* gender ambiguity, *T* testosterone, *DHT* dihydrotestosterone, *Post-hcg* laboratory values following human chorionic gonadotropin stimulation, *NA* not available

The basal T/DHT ratios in the cases (*n* = 17) ranged from 0.57–52.9 (median: 24.3). Baseline T/DHT values ranged from 5.3–52.9 in pubertal subjects. The posthCG stimulation T/DHT ratios (*n* = 6) ranged from 2.1–90 (median: 13.8). We noted that T/DHT could not be performed in the pubertal patients due to insurance coverage.

The Multidisciplinary Gender Assessment Committee has made a gender decision for 25 cases. Male gender assignment was made for 20/25 cases (80%), and a female decision was made (Cases 12 and 18) for 2/25 cases (8%) as a primary decision of the Council. One patient was lost to follow-up. The remaining 6 cases were planned for yearly re-evaluations for two main reasons: three parents from two separate families did not accept changing their sibling’s current sex assignment, and the remaining three children were too young for a comprehensive psychiatric evaluation regarding gender roles; a definitive assessment could not be made. However, to preserve testicular function, orchiopexy was performed, and annual follow-up evaluations were planned.

The two patients who were formerly assigned as females by the GAC were rediscussed, and according to redecision, one of them (Patient 12) decided to be supported as male. Patient 18 failed to attend the follow-up appointment. Gonadectomy was not recommended for any of these patients before the age of 18.

The parents of cases 1, 2 and 6 from family A refuted the assignment of the GAC toward the male gender, and these children were scheduled for serial evaluations. The follow-up of Patient 6 continued until the age of 18, and after the end of puberty, the GAC reassigned the patient as female. Cases 1 and 2 from Family A remain under consideration, as a conclusive decision has not been made due to the family’s vehement opposition to the male gender assignment (discussed separately). In the family’s history, Case 1 from Family A broke the Turkish record in pole vaulting, securing first place nationally and ranking 16th in the World Championship, whereas Case 6 of Family A achieved third place in Turkey in the shot put event.

#### Genetic data

The study group of patients (*n* = 29) was from 17 independent families, and all patients were shown to harbor *SRD5A2* pathogenic variants (see Table 2). The effects of the variants on external genitalia are presented in Table 3.

**Table 2:** Molecular profiles of *SRD5A2* (NM_000348.4) variants in our cases and corresponding literature references

Family	cDNA	Protein	Zygosity	dbSNP	MAF gnomAD/TR	CADD	ACMG (Varsome)	ClinVar ID	Reference (PMID^a^)
A, B, P	c.453delC	p.Leu152TyrfsTer8	Hom	-	0.000001 -	29.7	LP: PVS1, PM2	-	23664981
C	c.586G>A	p.Gly196Ser	Hom	rs121434250	0.000079 0.000595	25.5	P: PS1, PM5, PP5, PM1, PM2, PP3	3345	8110760
D, E, F, G, H,I	c.193G>C	p.Ala65Pro	Hom	-	0.000 -	22.7	LP: PP5, PM2, PP2	988403	20179965
J, K	c.164T>A	p.Leu55Gln	Hom	rs121434245	0.0000006 -	25.0	P: PP5, PM1, PM5, PM2, PP3	3339	8768837
L, R	c.269A>C	p.His90Pro	Hom	-	- -	26.4	VUS: PM1, PM2, PP3	-	35135181
M	c.542C>T	p.Pro181Leu	Hom	rs1057517829	0.000008 -	29.2	LP: PP5, PP3, PM2, PP2,	372520	8262007
N	c.13T>G	p.Cys5Gly	Het (m)	-	0.0000006 0.000204	25.6	VUS: PM2, PP2, PP3	-	30132287
O	c.282-2A>G	-	Het (m)	rs1340425455	0.000004 -	24.4	LP: PVS1, PM2	492900	12008688
	c.265C>G	p.Leu89Val	Hom	rs523349	0.696 0.731138	1.52	B: BA1, BP4, BP6, PM1	97400	11303586

*dbSNP* database of Single Nucleotide Polymorphisms, *gnomAD* genome aggregation database, *TRV* Turkish variome, *MAF* minor allele frequency, *CADD* combined annotation-dependent depletion, *ACMG* American college of medical genetics and genomics, *VUS* variant uncertain significance, *LP* likely pathogenic, *P* pathogenic, *B* benign, *PVS* pathogenic strong, *PM* pathogenic moderate, *PP* pathogenic supporting, *BA* benign strong, *BP* benign supporting, *Hom* homozygous, *Het* heterozygous, *m* maternal

^a^PubMed identifier number of the publication in which the relevant variant was reported

^-^absent

**Table 3:** Phenotypic features of *SRD5A2* (NM_000348.4) variants in our cases

cDNA	n	External Genitalia Score median (min-max)
c.193G>C	10	4.5 (1–8)
c.453delC	9	4.5 (1–9.5)
c.269A>C	4	4.13 (2.5–5)
c.164T>A	2	3.25 (3–3.5)
c.542C>T	1	2.5
c.13T>G	1	4.5
c.282-2A>G c.265C>G	1	6.5
c.586G>A	1	9

*n* number of patients harboring the variant

Segregation analyses were performed for all homozygous cases except for cases 7 and 14.

Two cases were shown to be heterozygous, whereas the rest of the study group had homozygous *SRD5A2* variants (93.1%). Among the heterozygous cases, patient 25 carried the heterozygous c.13T > G variant, and segregation analysis revealed the same heterozygous variant in the mother. The father of patient 25 did not declare any medical complaints and refused any genetic analysis regarding himself. In patient number 26, who carried the heterozygous c.282-2A > G variant, the father was wild-type, but the variant was found in the mother. Patient 26 also harbors a homozygous c.265C > G (p.Leu89Val) polymorphism, which is known to decrease 5-alpha reductase enzyme activity and was previously associated with micropenis [15].

Except for the father of Patient 18, heterozygous variants were found in both parents of all homozygous patients. Patient 18 had homozygous *SRD5A2* variants, whereas his father was shown to be WT for *SRD5A2*.

#### Family A

**Fig. 1:**
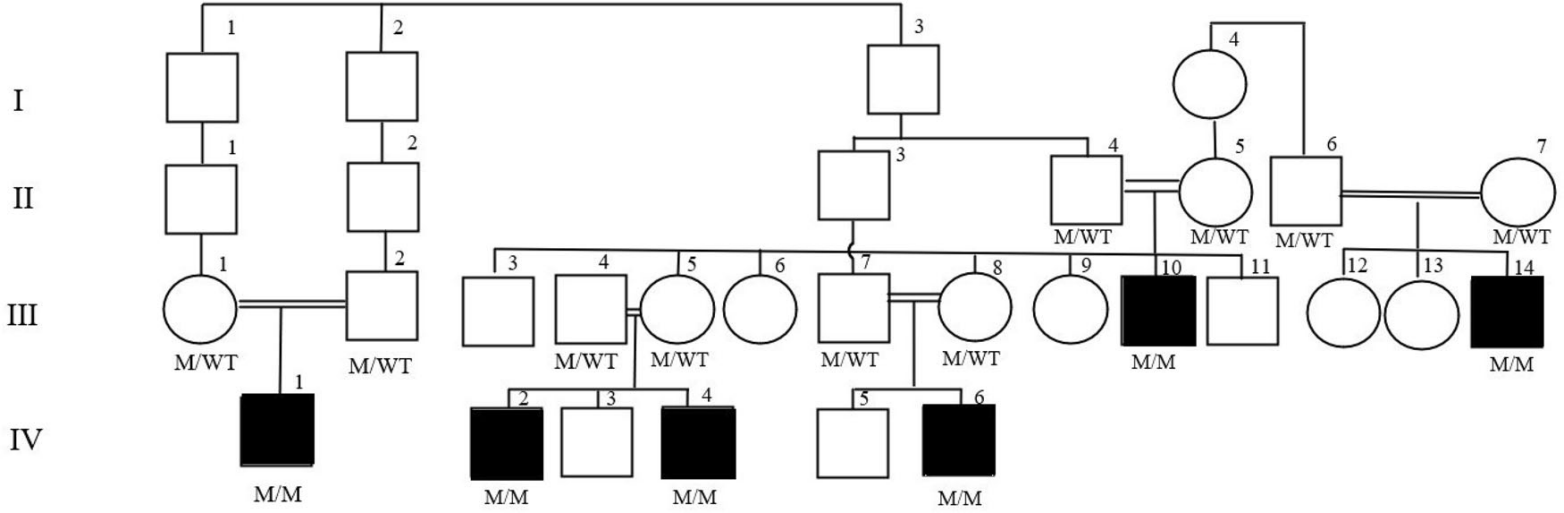
Pedigree of family A. The M and WT genotypes under each symbol represent the mutant and wild type, respectively. M/M homozygous pathogenic variant, M/WT heterozygous pathogenic variant

Three cases (III-10, IV-2, IV-4) from the same family (family A) warrant a separate heading (Fig. 1). The official gender of the participants upon admittance was female. The Multidisciplinary Gender Assessment Committee decided that male gender assignment was appropriate for all three cases. Contrary to the decision of the council toward male gender, the parents of III-10 refuted the male assignment. The follow-up of this patient continued until the age of 18, and after the end of puberty, Patient III-10 declared that she felt female. The Gender Assessment Committee reassigned the patient as female, and gonadectomy and associated surgeries were scheduled. Patient III-10 is currently a professional athlete with a female identity and has declared no gender dysphoria. We observed that the parents of cases IV-2 and IV-4, who had witnessed their relatives (cases III-10), had professional athletic careers and continued with them as females. The patients are still followed up in our clinic due to the risk of malignancy, and the prepubertal patients around the age of 18 are planning to be re-evaluated according to their final gender orientation.

## Discussion

The median age of the patients in our study was 71 months, which is lower than that reported in a French study, which reported a median age of 15 years [16]. While a neonatal diagnosis was reported in 29.7% of cases in an international study, none of our cases were diagnosed antenatal or neonatally [17]. However, with improvements in prenatal care and advancements in genetic examination opportunities, these patients may present at a much earlier age in the near future. Advancements in molecular genetics and the early identification of 5-alpha reductase deficiency might improve case management for sex determination in clinical practice.

In a European study including DSD patients with *SRD5A2* variants, the rate of consanguineous marriage was 37%, whereas it was 89.6% (26/29) within our study group [16]. A comprehensive study reported that as many as 69.1% of patients with *SRD5A2*-associated DSD have homozygous mutations [18]. In a study involving genetic diagnoses from our country, where consanguineous marriage rates are relatively high, all patients were reported to carry homozygous variants [19]. The elevated level of consanguinity within our cohort explains the observed prevalence of homozygous mutations compared with compound heterozygotes. We previously reported a similar situation in patients diagnosed with 21-hydroxylase deficiency [20]. Raising awareness about preventing future consanguineous marriages in families where the disease is concentrated may be useful. Additionally, preimplantation genetic diagnosis opportunities may be beneficial to consanguineous couples in families where cases are sequestered.

Approximately half of our patients had basal and posthCG stimulation T/DHT ratios below the reported cutoff values [21, 22]. This situation can be explained, in part, by residual 5α-R2 enzyme activity or 5α-R1 isozyme activity [23]. The literature suggests that this T/DHT ratio may lead to diagnostic confusion and is not essential [22]. For our patients, genetic analyses for *SRD5A2* were performed even where the T/DHT value was low, leading to a definitive genetic diagnosis. However, it is important to note that the sample size for which these values were reported was relatively small. In recent years, clinical findings supported by molecular investigations have gained prominence in establishing a diagnosis for our 46,XY DSD cases. We believe that, except in selected cases, routinely performing stimulated DHT/T analysis is unlikely to provide significant additional diagnostic value.

Most of our patients presented with ambiguous genitalia (51.7%). A study in China reported that 35.5% of patients diagnosed with 5α-reductase deficiency presented with micropenis [24]. The rate of presentation with micropenis in our cohort was lower (7.7%). Although a genotype‒phenotype relationship cannot be established, the most common p.Arg227Gln mutation, which was not present in any of our cases, was reported in a Chinese study [24]. Thus, our data may partially support the ethnic specificity reported for the gene.

Heterozygous variants were found in the parents of all the homozygous patients except for the father of Patient 18. Patient 18 had homozygous *SRD5A2* variants, whereas his father was shown to be WT for *SRD5A2*. In this case, a germline mutation of the father is one of the possible explanations. Another possibility is that the homozygous mutation could be a result of uniparental disomy, specifically unimaternal disomy. In this scenario, the child would have inherited two copies of the *SRD5A2* gene from the mother and none from the father.

In our study, the p.Ala65Pro variant, which we found in 10 patients, was the most common pathology, followed by p.Leu152fs*8, which we found in 9 patients. This finding was consistent with reports from our country [25]. The p.Gln126Arg variant was reported most frequently in an Italian study, but we did not encounter this variant in our patients [26]. In an Egyptian study, p.Gly34Arg and p.Gly196Ser were reported most frequently as homozygous variants in SDR5A2-related DSD cases [27]. The Arg246Gln variant, which is known to be present in various parts of the world, was not detected in our study, but p.Ala65Pro, which is reported to be specific to China, was detected at a high rate [17]. These results indicate that pathogenic variants in this gene may exhibit a significant degree of ethnic specificity.

In Patient 26 (Family O), a heterozygous c.282-2A > G variant in the SRD5A2 gene was identified, along with a homozygous c.265C > G polymorphism that partially reduces enzyme activity. The copresence of the c.282-2A > G variant, which is thought to affect splicing, and the c.265C > G polymorphism, which is linked to reduced enzymatic function, indicates the possibility of a combined impact on the overall activity of the SRD5A2 enzyme. This dual genetic alteration has been published, and this entity may have contributed to the clinical manifestations observed in this patient. Further investigation into the combined impact of other possible polymorphisms on SRD5A2-related pathologies is therefore warranted.

In line with current approaches, it was decided to support our cases predominantly toward a male identity. In an Italian study, gonadectomy was performed in 10 out of 24 patients diagnosed with 5α-reductase deficiency, whereas no gonadectomy was performed during childhood in our cohort [26]. Within Family 1, Case No. 6 was re-evaluated after the age of 18, a decision was made in favor of a female identity on the basis of psychiatric considerations, and gonadectomy and corrective surgeries were deemed appropriate. The follow-up of the other two cases from Family 1, whose parents denied raising them as males, continues. Gonadectomy or irreversible genital surgical procedures were not deemed appropriate/recommended for these two patients before puberty ended so that they reached an age where they could legally make their own sex decisions. In *SRD5A2* (and *HSD17B3*)-related pathologies, where virilization is often observed during puberty, the necessity and timing of irreversible interventions should be carefully considered [28].

Families in Middle Eastern countries, where the topic of gender roles can be taboo compared with those in first-world countries, can behave more conservatively. When early diagnosis and appropriate gender transition processes cannot be achieved, we observe that families do not comply with gender change decisions. In this case, it was observed that relatives, in particular, were influenced by each other and encouraged not to comply with medical recommendations. As we previously reported, we observed a similar phenomenon even in patients with 21-hydroxylase deficiency, a condition where sex assignment is typically more straightforward [29].

Patients with disorders of sex development (DSDs) and hyperandrogenism may continue to be identified as females for secondary gain. These gains may grant these individuals access to certain sports categories. However, is it ethical? Approximately 200 performance-associated polymorphisms have been identified that influence parameters such as mitochondrial capacity, body proportions, and lactic acid production in athletes from different disciplines. Since variables such as genetic polymorphisms, living conditions, race, and dietary habits affect athletes’ performance, some argue that fundamental equality cannot be achieved. However, no controlled study has examined the sole contribution of androgen levels to performance while all other factors are equal.

Prohibiting DSD individuals from participating in competitions could make them feel excluded from a field in which they find functional fulfillment. For example, Case 1 from Family A (during psychiatric evaluations) stated that she identifies herself more as an athlete than through her sexual identity. Individuals might also choose a female gender assignment by referencing other DSD individuals’ secondary benefits. For example, some relatives of Family A insisted on raising their other children as females, citing Cases 1 and 6 of Family A as references. Considering the complexity of the human subconscious and nature, it should be considered that the extent to which the gender selection decision is guided by secondary interests cannot be clearly determined.

In the future, a significant portion of successful competitors might consist of individuals with XY DSD, potentially negatively impacting other female athletes. It is reasonable to assume that with the increasing visibility of professional athletes with DSD, international authorities may introduce new regulations. According to a previous report, when hyperandrogenism is medically or surgically reduced to meet current competition limits, significant loss of muscle strength previously reported is not observed [30]. We noted that Patient 6 from Family A reported no performance loss two years after undergoing gonadectomy. However, our ability to predict the potential outcomes of possible forthcoming restrictions is limited.

This issue highlights a delicate balance: on the one hand, athletes with DSD deserve the right to compete and engage in sports freely; on the other hand, their presence in specific sports disciplines may inadvertently place cisgender female athletes at a competitive disadvantage, potentially skewing certain events. Furthermore, while these genetic advantages could support an athletic career for individuals with DSD, they may also possibly restrict opportunities due to imposed limitations or potential disqualification. This duality underscores the need for nuanced policies that respect the rights of all athletes while striving for equity and fairness in competitive sports. Ethical debates on this issue have persisted for over a century and seem to continue.

As a conclusion, in cases of *SRD5A2*-associated DSD even if a female sex assignment is made, irreversible interventions such as gonadectomy should be performed at the end of puberty. Some XY DSD patients may have difficulty adhering to medical team guidance during follow-up/treatment because of the negative impact on the patients and their families. Multidisciplinary committees should reevaluate prepubertal DSD cases on the basis of final opinions, and if necessary, they should be able to reconsider their decisions. Although early decisions are subject to ethical debate, it should not be forgotten that indecision can also have a detrimental effect on individuals.

## Data Availability

Genetic data details that support the findings of this study have been deposited in our Departments drive account. We may share it on demand through an e-mail contact.
